# Changes in vaginal cytokines concentrations during artificial insemination and natural service in beef heifers

**DOI:** 10.1186/s13104-024-06917-2

**Published:** 2024-10-14

**Authors:** Kyle J. McLean, Taylor B. Ault-Seay, Phillip R. Myer

**Affiliations:** Department of Animal Science, University of Tennessee Institution of Agriculture, Knoxville, TN USA

**Keywords:** Cytokines, Mating type, Pregnancy status, Reproductive environment, Vagina

## Abstract

**Objective:**

Heifer development is crucial for the optimization of reproductive efficiency in beef production. Heifer development is largely influenced by nutrition. Nutritional status of these heifers can influence immunological responses that are crucial for reproduction. Commercial Angus heifers (*n* = 9) were utilized, in a pilot study, to elucidate the effects of sampling time (days 0, 7, 14, 21, 28, and 35), pregnancy status, and type of mating on individual cytokine concentrations and cytokine profiles in the vagina following breeding.

**Results:**

Cytokine profiles were analyzed using MetaboAnalyst 5.0 and one-way ANOVAs were performed in R Studio to identify differences in individual cytokines based on sampling time, pregnancy status, and type of mating. Cytokine profiles were different (*P* = 0.05) 7 days after either mating type. Cytokines, IL-1β, IL-17a, MCP-1, and TNFα were different based on the mating type and pregnancy status. Multiple cytokines, IL-1α, IL-1β, IL-6, IL-8, IL-10, IL-17a, VEGFa, and MIP1α, were different between days regardless of pregnancy status. In conclusion, vaginal cytokines differ based on pregnancy status, type of mating, and time which may be indicative of vital pathways that need to be activated for pregnancy.

## Introduction

The livestock industry must increase sustainable production to provide enough nutrient-dense food to the anticipated 10 billion people by 2050, even as land availability decreases [1, 2]. Reproductive efficiency is highly dependent on proper development of heifers and will impact production efficiency in beef operations. Heifer development is largely influenced by nutrition [3, 4] and reproductive functions will suffer if nutrition is inadequate [5]. Often, developing females require good quality nutrient supplementation in ideally required amounts to ensure proper maturation. Nutritional status can also influence immunological responses of female reproductive tract [6], specifically, cytokines and angiogenic substrates that are crucial for reproductive processes. Therefore, increased inflammation caused by nutrient plane may affect reproductive functions through changes to the reproductive environment.

The embryo must utilize substrates within the uterine environment to develop until hemotrophic nutrient supply can be established.To prevent rejection, an immuno-tolerant uterine environment via cytokine signaling must be created to facilitate embryo attachment [7]. Cytokine networks complete a variety of roles in both reproductive and pregnancy related processes such as the estrous cycle [8], ovulation [9], embryonic development [10], and implantation [11]. Cytokines and chemokines have been reported to be important in inflammatory pathways for uterine remodeling and recruitment of immune cells during pregnancy, which differed between heifers of varying future reproductive success [12].Therefore, identifying when inflammatory signals become present within the reproductive tract could allow for alterations in management strategies to improve reproductive outcomes. It is also possible that these substrates could be used as biomarkers for viable pregnancies. Therefore, we hypothesized that cytokine and chemokine concentrations within the reproductive tract will differ based on pregnancy status and mating via artificial insemination or natural service.

## Methods

### Experimental design and sample collections

All experimental procedures involving animals were approved by the University of Tennessee Institutional Animal Care and Use Committee. Healthy, virgin 15 month old commercial Angus heifers (*n* = 9) from the fall calving herd housed in a single pen and located at the East Tennessee Research and Education Center (ETREC) were utilized to complete all objectives for the study. After completion of the study all animals were returned to the ETREC herd. Estrous synchronization was performed by the research center to synchronize the estrous cycle and day of breeding for all heifers. On the day of breeding (d 0), a vaginal flush was collected from every heifer. The vaginal flush was collected by placing 20 mL of sterile saline into the vagina via insertion of a two-way 4 Fr Foley catheter in the fornix vagina, mixed with vaginal fluid by rectal massage, removed through the catheter, and stored at -80 °C until cytokine quantification could be completed. A single artificial insemination was then performed by a single technician. Following the day of breeding, vaginal flushes were collected from every heifer on d 7, 14, 21, 28, and 35. On d 21 following sample collection, a fertile bull was introduced into the heifer group to service any heifers that did not become pregnant to the artificial insemination. Pregnancy diagnosis was completed via pregnancy-associated glycoprotein analyses [13] from blood samples collected on d 35 and 56 (35 days after the introduction of the bull). Cytokine concentrations of IL-1α, IL-1β, IL-10, IL-17a, IL-36ra, IL-8, IFN-γ, MCP-1, MIP-1α, MIP-1β, TNF-α, and VEGFa were quantified using the MILLIPLEX ^®^ MAP Bovine Cytokine/Chemokine Magnetic Bead Panel (MilliporeSigma, Burlington, MA, USA) according to manufacturer protocol, and analyzed on the Luminex 200 system (Luminex, Austin, TX, USA) at the University of Tennessee Institute of Agriculture Genomics Hub.

### Statistical analyses

Cytokine profile analyses were completed with log-transformed cytokine concentrations using MetaboAnalyst 5.0 [14]to identify differences in cytokine profiles within pregnancy status, type of mating, and day of sampling. The chemometrics analysis using orthogonal PLS-DA was utilized to evaluate cytokine profiles by week, type of mating, and pregnancy status. Following the identification of distinct cytokine profiles, ANOVA was conducted to elucidate differences in individual cytokines based on the mating type and pregnancy status on d 7 and d 28. A completely randomized design was implemented for individual statistical analyses in R Studio with the heifer as the experimental unit. One-way ANOVAs were performed via the aov function with fixed effects of sampling day, pregnancy status, and type of mating. Fisher’s LSD was utilized for mean separation. Means were reported different when *P* < 0.05 and tendencies at *P* < 0.10.

## Results

**Fig. 1:**
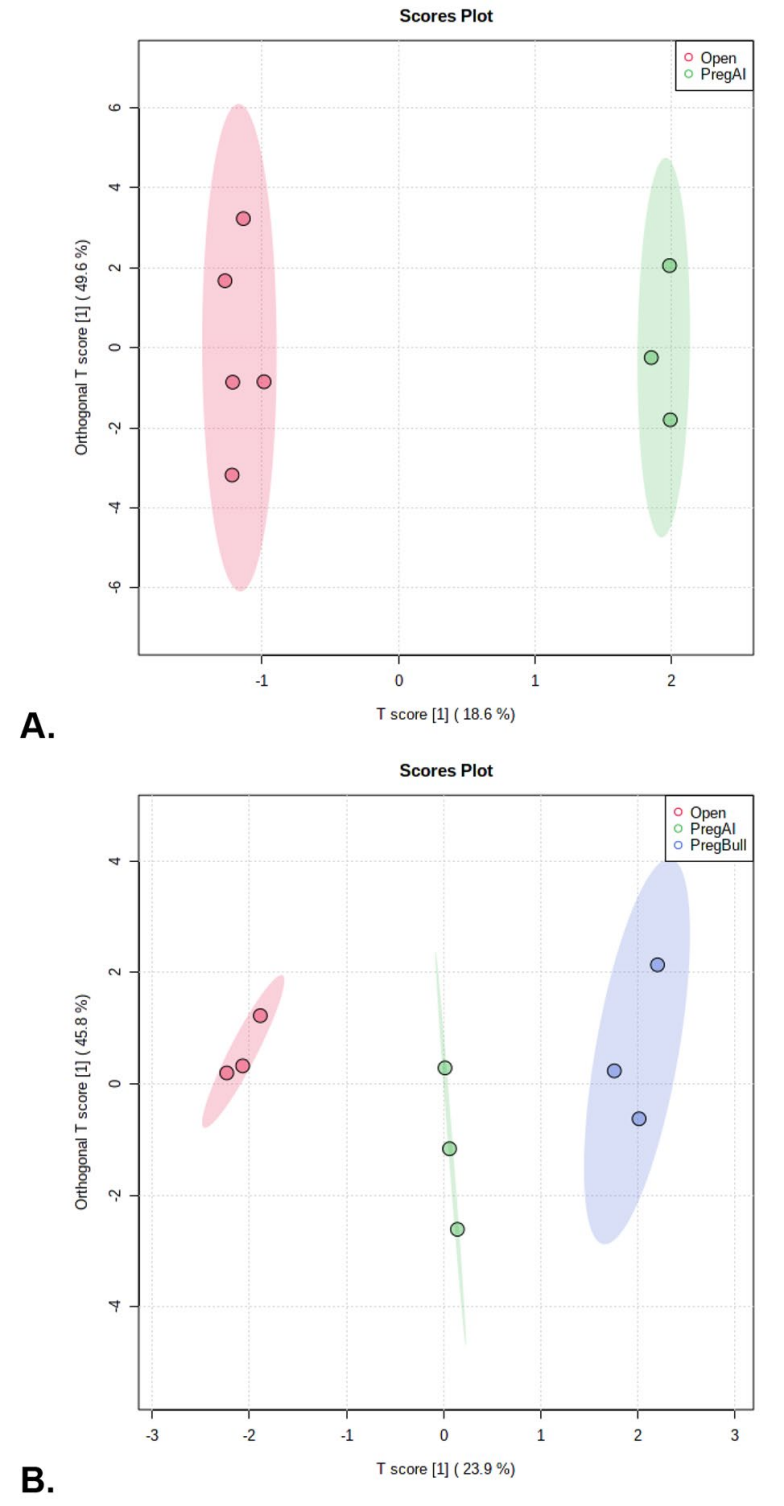
The impacts of pregnancy status and type of mating on vaginal cytokine profiles via PLS-DA during early gestation. **A)** Differences in cytokine profiles between non-pregnant (Open) and Pregnant via AI (PregAI) on d 7 of gestation. **B)** Differences in cytokine profiles between non-pregnant (Open), Pregnant via AI (PregAI)and Pregnant via natural service (PregBull)on d 28 following AI

Cytokine profile analyses revealed no differences (*P* > 0.10) in vaginal cytokine profiles between sampling days. However, orthogonal PLSDA identified distinct (*P* = 0.05) vaginal cytokine profiles between pregnant heifers from AI mating and non-pregnant (Open) heifers 7 days after AI (Fig. 1A). Distinct cytokine profiles were also found between AI bred heifers, bull-bred heifers, and non-pregnant (open) heifers 7 days after the introduction of the bull (Fig. 1B). No differences (*P* > 0.10) were found between individual cytokines on d 7. However, IL-1β, IL-17a, MCP-1, and TNFα were different based on the mating type and pregnancy status on d 28 (Table 1). All cytokines were increased in the bull-bred heifers (*P* ≤ 0.05). No differences were found in IL-1β or IL-17a concentrations between AI-bred or non-pregnant heifers (*P* > 0.10). Concentrations of MCP-1 were lowest in non-pregnant heifers and intermediate in AI-bred heifers compared to bull-bred heifers (*P* = 0.04). The vaginal concentrations of TNFα were lowest in AI-bred heifers and intermediate in non-pregnant heifers compared with bull-bred heifers (*P* = 0.05). To elucidate differences in mating type a direct comparison between bull-bred and AI-bred heifers 7 d after mating was conducted (Table 1). Interferon-γ (*P* = 0.05), IL-17a (*P* = 0.01), and TNFα (*P* = 0.02) were all found to be increased by 7 d after a natural mating (bull-bred) compared with a pregnancy resulting from AI mating. There was also a tendency (*P* = 0.08) for IL-36ra to be increased in natural mating compared with AI mating. Even without distinct cytokine profiles from PLS-DA between sampling days, multiple cytokines, IL-1α, IL-1β, IL-6, IL-8, IL-10, IL-17a, VEGFa, and MIP1α, were found to be different over the course of the study regardless of pregnancy status (Table 2). Interleukin-8 (*P* = 0.06), IL-17a (*P* = 0.08), and MIP1α (*P* = 0.09) only tended to be different. All other cytokines, IL-1α (*P* = 0.01), IL-1β (*P* = 0.01), IL-6 (*P* = 0.03), IL-10 (*P* = 0.03), and VEGFa (*P* = 0.01) were greatest prior to AI on d 0 and lowest on d 35 with d 7 to 28 fluctuating dependent on cytokine (Table 2).

**Table 1:** The effects of pregnancy status and type of mating on individual cytokine concentrations with the vaginal fluid of beef heifers

Cytokine	Open	Bull Bred (d7)	A.I. Bred (d 35)	SEM	*P* Value
IL-1β	1.24	8.09	1.50	2.02	0.05
IL-17a	0.60^b^	2.32^a^	0.68^b^	0.29	0.01
MCP-1	19.8^a^	990.4^b^	110.6^ab^	278.0	0.04
TNFα	4.39^ab^	18.09^b^	1.15^b^	2.97	0.05
---	---	Bull Bred (d7)	A.I. Bred (d 7)	SEM	*P* Value
IFNγ	---	3.86^a^	0.86^b^	0.76	0.05
IL17a	---	2.32^a^	0.47^b^	0.31	0.01
IL36ra	---	13.19	3.50	2.99	0.08
TNFa	---	18.09^a^	1.93^b^	2.89	0.02

^a, b^Means without a common superscript differ *P* < 0.05

**Table 2:** The effects of time after Estrus synchronization on individual cytokine concentration with the vaginal fluid of beef heifers

Cytokine	Day 0	Day 7	Day 14	Day 21	Day 28	Day 35	SE	*P* Value
IL-1α	198.2^a^	59.2^b^	108.4^ab^	25.4^b^	57.5^b^	19.4^b^	46.0	0.01
IL-1β	29.2^a^	4.8^b^	23.3^ab^	3.6^b^	3.6^b^	4.8^b^	7.1	0.01
IL-6	17.8^a^	3.3^b^	7.5^ab^	1.1^b^	1.2^b^	3.5^b^	4.5	0.03
IL-8	315.5^a^	195.4^ab^	236.28^ab^	62.6^b^	223.4^ab^	59.0^b^	87.4	0.06
IL-10	49.3^a^	12.7^b^	43.0^ab^	16.9^ab^	13.0^b^	12.8^b^	11.0	0.03
IL-17a	14.2	1.84	6.72	1.60	1.20	1.64	4.95	0.08
VEGFa	710.6^a^	157.6^b^	207.9^b^	77.9^b^	258.4^b^	47.7^b^	138.7	0.01
MIP1a	27.76	11.55	18.49	11.75	13.61	12.18	5.72	0.09

^a, b^Means without a common superscript differ *P* < 0.05

## Discussion

Cytokines are essential for successful pregnancy establishment and retention through the promotion of events for attachment. Most research has focused on systemic and uterine cytokines, thus, making these data novel and insightful to the inflammatory changes within the vaginal environment that may initiate necessary alterations for early gestation. Research has also indicated that systemic cytokines may fluctuate by stage of the estrous cycle [8, 15, 16], potentially in preparation for gestation. Within the endometrium of heifers, the gene expression of IL-1β and IFN-γ, but not IL-1α, IL-6, and IL-10, were affected by the stage of the estrous cycle [15]. The current study found changes over time of IL-1α, IL-1β, IL-6, IL-8, IL-10, IL-17a, VEGFa, and MIP1α. These cytokines were greatest following an estrus synchronization protocol and intermediate on day 21 which would correspond to a natural estrus in non-pregnant animals. Thus, agreeing and expanding on data previously reported [15]. During pregnancy, IL-10 expression by endometrial immune cells was increased compared to cyclic heifers [17] and goats [18]. While IL-10 was not identified as different amongst open, bull-bred, and AI-bred. TNFα was greater in both the 3-way and direct (bull vs. AI) comparison 7 days after mating indicating the initiation of an inflammatory response. Ault-Seay et al., [16] reported IL-10 concentration to follow a similar pattern to that of TNFα in developing heifers. Therefore, the time between samples in the current dataset may not have been frequent enough to capture the impacts of IL-10. Another potential explanation is that IL-36ra, which is anti-inflammatory [21], may have a larger role in modulating the inflammatory status of the reproductive tract during early gestation of cattle. Furthermore, classical functions of TNFα have been reported in the activation of macrophages and overall inflammation through interactions with IL-1α, IL-1β, IL-8, and IL-6 [19, 20]. All of these were found in different concentrations either by pregnancy status, mating type, or sampling day. Additionally, a collaborative effect on physiological function was reported in cows that failed to rebreed. These cows had a higher ratio of IL-1α and IL-1β to IL-10 than those that became pregnant [22]. In this dataset, only IL-1β was observed as different in pregnancy status analyses. However, this may only be indicative of a stronger role or a quicker response of IL-1β and TNFα compared with IL-10 and IL-1α. Previous work in our lab showed IL-1α to be relatively stable during heifer development [16]. Further indicating a more indirect role of this cytokine in reproduction. Monocyte chemoattractant protein-1 was differentially expressed in the current dataset. Monocyte chemoattractant protein-1 regulates cell migration and infiltration into tissue and has been shown to be important in macrophage accumulation during murine pregnancy [23] as well as during ovine embryonic attachment and placentation [24]. Since MCP-1 was determined to be different between open, bull-bred, and AI-bred heifers but not in a direct comparison between mating types, this may indicate that MCP-1 is influential in bovine pregnancy and reproductive tract inflammation regardless of where semen is deposited. Interestingly, IFN-γ was found to be different between mating types but not in pregnancy status which is somewhat contradictory to other research but could be a function of where the samples were collected. Interferon-γ expression was lower in the endometrium of heifers that had viable embryos recovered on day 7, compared to heifers with viable non-embryos [25]. The increased concentrations of IFN-γ in the heifers who were pregnant from mating with the bull is most likely a direct connection with the classical functions of IFN-γ for a pro-inflammatory response and recruitment of immune cells to fight pathogens. This could also be a tissue-specific difference between the vagina and uterus during early gestation. The presence of these pro-inflammatory cytokines, IL-1α, IL-1β,IFN-γ, MCP-1, and TNFα, in the reproductive tract is typically associated with the involution and postpartum disease. Interestingly, cows that developed endometritis had a reduction in pro-inflammatory cytokines earlier in the postpartum period than cows that did not develop endometritis [26]. However, in the current study, it appears that immune signaling occurs differently in successful insemination but even more so when that insemination occurs in the vagina in natural mating compared with deposition in the uterus as it is in artificial insemination. Natural service mating in the current study (i.e. vaginal insemination), is likely driving the difference observed due to proximity to sample collection and the known promotion of cytokine synthesis within the reproductive tract from the active components within the seminal fluid [27]. These components cooperate with the epithelial cells to facilitate embryo tolerance, expansion, and implantation in mammals [27]. However, this makes the differences observed for MCP-1 and TNFα even more intriguing. Another important function of cytokines during early gestation is vascular modulation. The uterus, ovaries, and placenta exhibit regular intervals of rapid growth with high vascularity and blood flow [28]. Progesterone has been reported to influence IL-36ra and VEGFa [16] which supports findings that progesterone appears to be a primary regulator of uterine vascular function in mammals [29] and potentially the onset of a quiescent uterine environment. However, during heifer development, the angiogenic cytokine IL-17a was reported a negative trend over time potentially indicative of sufficient blood supply to support physiological function [16]. The idea of IL-17a increasing in preparation for the required increase in blood flow would agree with this data where IL-17a increased in bull bred heifers 7 days after insemination. However, the AI bred heifers did not exhibit this increase which may be a difference in insemination location or a potential pathway that is not adequately stimulated when copulation does not occur or seminal plasma is diluted as is the case with AI. The importance of VEGFa during early gestation is well-documented in multiple species [30, 31]. In the current study, VEGFa was not influenced by pregnancy status or type of mating but was different across sampling days. This may be indicative of changes in the reproductive tract following ovulation in preparation for a potential pregnancy. In conclusion, vaginal cytokines and cytokine profiles differ based on pregnancy status, type of mating, and time following ovulation. However, more research needs to be conducted to elucidate the importance of these changes and the mechanisms for establishment of pregnancy.

## Limitations

The major limitation of this study is the low number of animals in each pregnancy category, only 3 for open, AI bred, and bull bred. Additionally, a more direct comparison between uterine and vaginal environment would be beneficial.

## Data Availability

Data is available upon request from the corresponding author.

## Abbreviations

AI: Artificial Insemination
ANOVA: Analysis of Variance
IL: Interleukin
IFN: Interferon
MCP: Monocyte Chemoattractant Protein
MIP: Macrophage Inflammatory Protein
PLS-DA: Partial Least Squares-Discriminant Analysis
TNF: Tumor Necrosis Factor
VEGF: Vascular Endothelial Growth Factor

## References

[1] Elliot I. Meat output must double by 2050. Feedstuffs. http://feedstuffsfoodlink.com/story-meat-output-must-double-by-2050-71-66920. Accessed 1/15/2013.

[2] Reynolds LP, Wulster-Radcliffe MC, Aaron DK, Davis TA. Importance of animals in agricultural sustainability and food security. J Nutr. 2015;145(7):1377–9. 10.3945/jn.115.212217. 25972529 10.3945/jn.115.212217 PMC6625004

[3] Freetly HC, Kuehn LA, Cundiff LV. Growth curves of crossbred cows sired by Hereford, Angus, Belgian Blue, Brahman, Boran, and Tuli bulls, and the fraction of mature body weight and height at puberty. J Anim Sci. 2011;89(8):2373–9. 10.2527/jas.2011-3847. 21531851 10.2527/jas.2011-3847

[4] Perry GA. Factors affecting puberty in replacement beef heifers. Theriogenology. 2016;86(1):373–8. 10.1016/j.theriogenology.2016.04.051. 27160450 10.1016/j.theriogenology.2016.04.051

[5] Short RE, Bellows RA, Staigmiller R, Berardinelli J, Custer E. Physiological mechanisms controlling anestrus and infertility in postpartum beef cattle. J Anim Sci. 1990;68(3):799–816. 10.2527/1990.683799x. 2180877 10.2527/1990.683799x

[6] Chandra RK. Nutrition and the immune system: an introduction. Am J Clin Nutr. 1997;66(2):S460–3. 10.1093/ajcn/66.2.460S. 10.1093/ajcn/66.2.460S 9250133

[7] Schjenken JE, Tolosa JM, Paul JW, Clifton VL, Smith RJ. Mechanisms of maternal immune tolerance during pregnancy. Recent Adv Res Hum Placenta. 2012;11:211–42.

[8] Krakowski L, Zdzisinska B. Selected cytokines and acute phase proteins in heifers during the ovarian cycle course and in different pregnancy periods. J Bulletin-Veterinary Inst Pulawy. 2007;51(1):31.

[9] Espey LL, Bellinger AS, Healy JA. Chapter 9 - ovulation: an inflammatory cascade of gene expression. In: Leung PCK, Adashi EY, editors. The Ovary (Second Edition). San Diego: Academic; 2004. pp. 145–65.

[10] Zolti M, Ben-Rafael Z, Meirom R, Shemesh M, Bider D, Mashiach S, Apte RN. Cytokine involvement in oocytes and early embryos. Fertil Steril. 1991;56(2):265–72. 10.1016/s0015-0282(16)54483-5. 2070856 10.1016/s0015-0282(16)54483-5

[11] Simón C, Moreno C, Remohí J, Pellicer A. Cytokines and embryo implantation. J Reprod Immunol. 1998;39(1):117–31. 10.1016/S0165-0378(98)00017-5. 9786457 10.1016/s0165-0378(98)00017-5

[12] Killeen AP, Morris DG, Kenny DA, Mullen MP, Diskin MG, Waters SM. Global gene expression in endometrium of high and low fertility heifers during the mid-luteal phase of the estrous cycle. BMC Genom. 2014;15(1):234. 10.1186/1471-2164-15-234. 10.1186/1471-2164-15-234 PMC3986929 24669966

[13] Pohler KG, Pereira MHC, Lopes FR, Lawrence JC, Keisler DH, Smith MF, Vasconcelos JLM, Green JA. Circulating concentrations of bovine pregnancy-associated glycoproteins and late embryonic mortality in lactating dairy herds. J Dairy Sci. 2016;99(2):1584–94. 10.3168/jds.2015-10192. 26709163 10.3168/jds.2015-10192

[14] Pang Z, Chong J, Zhou G, de Lima Morais DA, Chang L, Barrette M, Gauthier C, Jacques PÉ, Li S, Xia J. MetaboAnalyst 5.0: narrowing the gap between raw spectra and functional insights. Nucleic Acids Res. 2021;49:W388–96. 10.1093/nar/gkab382. 34019663 10.1093/nar/gkab382 PMC8265181

[15] Oliveira LJ, Mansourri-Attia N, Fahey AG, Browne J, Forde N, Roche JF, Lonergan P, Fair T. Characterization of the Th Profile of the bovine endometrium during the Oestrous cycle and early pregnancy. PLoS ONE. 2013;8(10):e75571. 10.1371/journal.pone.0075571. 24204576 10.1371/journal.pone.0075571 PMC3808391

[16] Ault-Seay TB, Harrison TD, Brandt KJ, Payton RR, Schneider LG, Myer PR, Rhinehart JD, Rispoli LA. and K. J. McLean. 2021 the effects of protein level on cytokines and chemokines in the uterine environment of beef heifers during development. J Anim Sci 99:skab105. 10.1093/jas/skab105 10.1093/jas/skab105 PMC8188814 33822060

[17] Vasudevan S, Kamat MM, Walusimbi SS, Pate JL, Ott TL. Effects of early pregnancy on uterine lymphocytes and endometrial expression of immune-regulatory molecules in dairy heifers. Biol Reprod. 2017;97(1):104–18. 10.1093/biolre/iox061. 28633489 10.1093/biolre/iox061

[18] Imakawa K, Nagaoka K, Nojima H, Hara Y, Christenson RK. 2005. Changes in immune cell distribution and IL-10 production are regulated through endometrial IP-10 expression in the goat uterus. 53(1):54–64. 10.1111/j.1600-0897.2004.00243.x 10.1111/j.1600-0897.2004.00243.x 15667526

[19] Maini RN, Elliott MJ, Brennan FM, Feldmann M. Beneficial effects of tumour necrosis factor-alpha (TNF-alpha) blockade in rheumatoid arthritis (RA). Clin Exp Immunol. 1995;101(2):207–12. 10.1111/j.1365-2249.1995.tb08340.x. 7648705 10.1111/j.1365-2249.1995.tb08340.x PMC1553280

[20] Parameswaran N, Patial S. Tumor necrosis factor-α signaling in macrophages. Crit Rev Eukaryot Gene Expr. 2010;20(2):87–103. 10.1615/critreveukargeneexpr.v20.i2.10. 21133840 10.1615/critreveukargeneexpr.v20.i2.10 PMC3066460

[21] Queen D, Ediriweera C, Liu L. Function and regulation of IL-36 signaling in inflammatory diseases and cancer development. Front Cell Dev Biol. 2019;7. 0.3389/fcell.2019.00317. 10.3389/fcell.2019.00317 PMC6904269 31867327

[22] Herath S, Lilly ST, Santos NR, Gilbert RO, Goetze L, Bryant CE, White JO, Cronin J, Sheldon IM. Expression of genes associated with immunity in the endometrium of cattle with disparate postpartum uterine disease and fertility. Reprod Biol Endocrin. 2009;7(1):55. 10.1186/1477-7827-7-55. 10.1186/1477-7827-7-55 PMC2702306 19476661

[23] Wood GW, Hausmann EH, Kanakaraj K. Expression and regulation of chemokine genes in the mouse uterus during pregnancy. Cytokine. 1999;11:1038–45. 10.1006/cyto.1999.0513. 10623429 10.1006/cyto.1999.0513

[24] Asselin E, Johnson GA, Spencer TE, Bazer FW. Monocyte chemotactic protein-1 and – 2 messenger ribonucleic acids in the ovine uterus: regulation by pregnancy, progesterone, and interferon-tau. Biol Reprod. 2001;64:992–1000. 10.1095/biolreprod64.3.992. 11207217 10.1095/biolreprod64.3.992

[25] Beltman ME, Forde N, Lonergan P, Crowe M. Altered endometrial immune gene expression in beef heifers with retarded embryos. Reprod Fertil Dev. 2013;25(6):966–70. 10.1071/RD12232. 23034328 10.1071/RD12232

[26] Galvão KN, Santos NR, Galvão JS, Gilbert RO. Association between Endometritis and endometrial cytokine expression in postpartum holstein cows. Theriogenology. 2011;76(2):290–9. 10.1016/j.theriogenology.2011.02.006. 21496904 10.1016/j.theriogenology.2011.02.006

[27] Robertson SA. Seminal plasma and male factor signaling in the female reproductive tract. Cell Tissue Res. 2005;322:43–52. 10.1007/s00441-005-1127-3. 15909166 10.1007/s00441-005-1127-3

[28] Reynolds LP, Grazul-Bilska AT, Redmer DA. Angiogenesis in the female reproductive organs: pathological implications. Int J Exp Pathol. 2002;83(4):151–63. 10.1046/j.1365-2613.2002.00277.x. 12485460 10.1046/j.1365-2613.2002.00277.x PMC2517679

[29] Murray P, Wynn T. Protective and pathogenic functions of macrophage subsets. Nat Rev Immunol. 2011;11723–737. 10.1038/nri3073. 10.1038/nri3073 PMC3422549 21997792

[30] Pfarrer CD, Ruziwa SD, Winther H, Callesen H, Leiser R, Schams D, Dantzer V. Localization of vascular endothelial growth factor (VEGF) and its receptors VEGFR-1 and VEGFR-2 in bovine placentomes from Implantation until term. Placenta. 2006;27(8):889–98. 10.1016/j.placenta.2005.09.004. 16263165 10.1016/j.placenta.2005.09.004

[31] McLean KJ, Crouse MS, Crosswhite MR, Pereira NN, Dahlen CR, Borowicz PP, Reynolds LP, Ward AK, Neville BW, Caton JS. Impacts of maternal nutrition on uterine and placental vascularity and mRNA expression of angiogenic factors during the establishment of pregnancy in beef heifers. Translational Anim Sci. 2017;1(2):160–7. 10.2527/tas2017.0019. 10.2527/tas2017.0019 PMC7205333 32704639

